# Problem gaming and suicidality: A systematic literature review

**DOI:** 10.1016/j.abrep.2022.100419

**Published:** 2022-03-11

**Authors:** Eilin K. Erevik, Helene Landrø, Åse L. Mattson, Joakim H. Kristensen, Puneet Kaur, Ståle Pallesen

**Affiliations:** a Department of Psychosocial Science, University of Bergen, Bergen, Norway; b Resource Centre on Violence, Traumatic Stress and Suicide Prevention, Haukeland University Hospital, Bergen, Norway

**Keywords:** Gaming disorder, Gaming addiction, Self-injurious behaviour, Self-harm, Suicide plans

## Abstract

**Background:**

No studies have so far synthesised the current evidence concerning a possible relationship between problem gaming and suicidality. We therefore conducted a systematic review of the literature. Our objective was to investigate the relationship between problem gaming and suicidality. The review was funded by the Norwegian Competence Center for Gambling and Gaming Research.

**Methods:**

The review was pre-registered in PROSPERO International prospective register of systematic reviews (CRD42021279774). Searches were conducted in Web of Science, PsycINFO, EMBASE, PubMed and Google Scholar, September 2021. Studies that reported data on the relationship between problem gaming and suicidality, published between 2000 and 2021, and written in any European language were included. Studies investigating internet addiction/problematic internet use and not problem gaming, specifically, and studies investigating mental health in general or mental health outcomes other than suicidality, were excluded. Data from the included studies were extracted independently by two coders who also evaluated for risk of bias using the Newcastle-Ottawa Quality Assessment Scale. The results from each included study were presented in a table.

**Results:**

A total of 12 cross-sectional studies, with in total 88,732 participants, were included in the review. In total 10 studies investigated the association between problem gaming and suicidal ideation. One of these also investigated the association between problem gaming and suicide attempts. Two studies combined suicidal ideation and suicide attempts into one variable and investigated the association between that variable and problem gaming. In total 11 of the 12 included studies found positive, crude associations between problem gaming and suicidal ideation/attempts. Five studies adjusted for possible confounding variables. Three of these still found significant associations between problem gaming and suicidal ideation, one found a positive but not statistically significant association, and the fifth found an inverse, non-significant association.

**Discussion:**

The current findings indicate that there is an association between problem gaming and suicidal ideation, and likely between problem gaming and suicide attempts. The most important limitation of the included studies is the lack of longitudinal designs. Future studies should aim to investigate the causality and mechanisms in the relationships using more stringent designs.

## Highlights

• This is the first review on the association between problem gaming and suicidality.
• 12 studies were identified, all of which found a positive association.
• Future studies should investigate the causality and mechanisms in the relationship.

## 1 Introduction

During the last decades gaming has become a common leisure activity that more and more people of different ages and genders engage in (Gilbert, 2021, Pallesen et al., 2020). Although gaming for most is a fun and recreational activity, a small minority is expected to experience problems related to their gaming, for instance conflicts with close ones or impaired physical and/or mental health (Delfabbro et al., 2021, Krossbakken et al., 2018, Pallesen et al., 2020, Stevens et al., 2021). The latter has been recognized in modern psychiatric nosology as “internet gaming disorder” and “gaming disorder” in the fifth edition of the Diagnostic and Statistical Manual of Mental Disorders (DSM-5, American Psychiatric Association, 2013) and in the eleventh edition of the International Classification of Diseases (ICD-11, World Health Organization, 2019), respectively. Problem gaming is a wider term, used to describe individuals who experience one or more problems related to their gaming. It should be noted that both the gaming-related diagnoses and the real existence of problem gaming in general, are controversial (Carras and Kardefelt-Winther, 2018, van Rooij et al., 2018). Among other things, it has been argued that the directionality between problem gaming and associated problems (e.g., depression) is unclear and that employing an addiction-framework on gaming is unwarranted (Carras and Kardefelt-Winther, 2018, van Rooij et al., 2018). The controversies and uncertainty concerning problem gaming underscore the importance of investigating potential antecedents, covariates, and consequences of this entity. Suicidality is a highly worrisome and serious outcome, which might be related to problem gaming (e.g., Khalil et al., 2020).

Suicidality reflects suicidal ideation, suicide attempts and suicide which are all characterised by a desire to die. However, the three concepts also have important differences, and there are also important differences within these outcomes (e.g., in the type of suicidal ideation experienced) (Klonsky et al., 2016, Meyer et al., 2010). Suicidal ideation can be defined as “thinking about, considering, or planning suicide”, whereas suicide attempts represent “nonfatal, self-directed, potentially injurious behaviour with an intent to die as a result of the behaviour”. Suicide construes “death caused by self-directed injurious behaviour with an intent to die as a result of the behaviour” (Klonsky et al., 2016, p. 309). All these outcomes involve a great deal of harm and distress for the affected individual and those close to them, and are costly for society (Ingabire and Richters, 2020, Klonsky et al., 2016, O’Dea and Tucker, 2005, van Spijker et al., 2012, van Spijker et al., 2011). Hence, reducing the prevalence rates of suicidality is an important public health matter (World Health Organization, 2014).

Problem gaming can be hypothesised to be associated with both suicidal ideation, suicide attempts, and suicide, in which several causal pathways and mechanisms may be involved. For one, problem gaming may cause suicidality. One pathway through which problem gaming might be speculated to increase the likelihood of suicidality is through increasing psychological distress and impulsivity which in turn may increase the likelihood of suicidality (Krossbakken et al., 2018, Klonsky et al., 2016, Şalvarlı and Griffiths, 2019). Further, it is reasonable to expect that individuals who experience problem gaming will use more time on gaming than most other gamers (Brunborg et al., 2013). Because of this they might be more exposed to elements in video games that may heighten the likelihood of suicidality for example cyberbullying and violence (Brailovskaia et al., 2018, Förtsch et al., 2021, Huang et al., 2019). However, it is important to note that the claim that playing violent video games may increase the incidences of real-world violence is controversial. Many would argue that the claim is not supported by the current evidence base (Ferguson et al., 2021). In addition to problem gaming possibly causing suicidality, the reversed causal pathway may also be at play. It can be speculated that some individuals who experience suicidal ideation, or have attempted suicide, may seek video games as a way of escaping from the distress they experience. Finally, problem gaming and suicidality might be associated in part because of common third variables predicting both problem gaming and suicidality (e.g., gender, depression) (Brunborg et al., 2013, Klonsky et al., 2016, Krossbakken et al., 2018). One potential third variable that may be particularly relevant is escapism as both problem gaming and suicidality have been suggested, and found, to be associated with a motive of escaping one‘s reality (Baumeister, 1990, Demetrovics et al., 2011, Landrault et al., 2020, Montag et al., 2019). Another class of potential third variables are personality traits, in which both problem gaming and suicidality have been found to positively associated with neuroticism and inversely associated with extroversion and conscientiousness (Akbari et al., 2021, Blüml et al., 2013, Brezo et al., 2006).

### 1.1 Rationale for the current study

Investigating the potential relationship between problem gaming and suicidality may illuminate the ontology of problem gaming in which longitudinal designs and adjustment for relevant third variables may give indications on the causality in the potential relationship. In addition, investigating the potential relationship between problem gaming and suicidality might inform policymakers and clinicians who work with minimising potential harm related to problem gaming and/or preventing suicidality. Clinical and preventive recommendations should always be based on the best and most comprehensive scientific information, in which systematic reviews are regarded as the best sources (Clarke & Horton, 2001). To the best of our knowledge, there currently do not exist any systematic review investigating the relationship between problem gaming and suicidality. Hence, we conducted a systematic review to investigate if there is an association between problem gaming and suicidality, the strength of this possible association, and whether there are some indications concerning the mechanisms and causality at play in this possible association.

## 2 Methods

### 2.1 Search strategy and inclusion criteria

The literature review was performed according to the guidelines of the Preferred Reporting Items for Systematic Reviews and Meta-Analyses (PRISMA) (Page et al., 2021). The review was pre-registered in PROSPERO International prospective register of systematic reviews (CRD42021279774). It was stated in the protocol that the database Open Grey would be searched. Such searches were not conducted due to oversight. Further, it was also stated in the protocol that the association between gaming in general (i.e., not limited to problem gaming) and suicidality would be investigated. However, only studies investigating the relationship between problem gaming and suicidality was included in the current study. We decided to limit our investigation to problem gaming because we consider this exposure to be more relevant for clinical practice and because we wanted to avoid pathologizing a common leisure activity (i.e., gaming). Systematic electronic literature searches were conducted on the 30th of September 2021 in the databases Web of Science (Core Collection), APA PsycINFO, EMBASE, and PubMed (MEDLINE). These databases were chosen as they are among the largest databases for research in psychology, health, and medicine. Additional searches were made in Google Scholar and by inspecting the reference lists of included articles.

The keywords used in the structured search are displayed in Table 1. The same search strategy was used in all the databases. No MeSH or other expanders were used in the searches.

**Table 1:** Search Keywords.

	**Problem gaming keywords**	**Operand**	**Suicide ideation, suicide attempts, and suicide keywords**
	“Game*”		
	“Gaming”		
OR	“Videogaming”	AND	“Suicid*”
	“Videogame*”		
	“Esport*”		
*Truncation.			

### 2.2 Selection

The following inclusion criteria were employed: 1) Peer-reviewed articles that report data on the relationship between problem gaming and suicidality, 2) articles published from the year 2000 to the time of the search, and 3) articles in any European language. Problem gaming was conceptualised as any gaming pattern that the authors of the specific article deemed problematic, excessive, pathological or similar. The exclusion criteria were: 1) Articles that only look at internet addiction/problematic internet use without looking at problem gaming in isolation, and 2) articles that only look at mental health in general or mental health outcomes other than suicidality. The literature search and the selection of studies were conducted in parallel and independently by two scholars (names omitted for anonymous review).

### 2.3 Data extraction and quality assessment

The process of data extraction was also conducted in line with PRISMA guidelines (Page et al., 2021). To extract the relevant data from the included studies, an extraction form was developed to register and code relevant information about the studies. The form was used to extract data on the following variables; authors and year, study design, country and continent, sample size, proportion of men, from where the sample was recruited, how problem gaming was measured (i.e., as a continuous or categorical outcome), specific measurement instrument used to measure problem gaming, how suicidality were measured (i.e., as a continuous or categorical outcome), approach used to measure/identify suicidality, which confounders that were controlled for, proportion of problem gamers, type of estimate, the reported empirical association between problem gaming and suicidal ideation, suicide attempts and/or suicide, *p*-value, and confidence interval. All types of effect estimates were extracted. After the extraction it was clear that many of the studies reported Odds Ratio (*OR*) or enough data to calculate *OR*. Hence, in the presentation of the results *OR*s were presented as an effect estimate in the instances where *OR* was available or could be calculated. No attempts were made to extract or collect missing or unclear information. The results from the data extraction were presented in a table where the following information from each included study were presented: Authors (year), country, sample size, proportion of men in the sample, mean and standard deviation for age, proportion of individuals with problem gaming in the sample, suicidality outcome (i.e., ideation, attempts, or completed suicides), and main findings (i.e., effect estimate, confidence interval, *p*-value, and which if any covariates that were adjusted for). We chose to present and synthesise the results through a table instead of a meta-analysis because the included studies varied in terms of how they measured problem gaming and suicidality and we therefore thought it would be erroneously to calculate a common effect size. The outcomes which we chose to include in the table was chosen because we deemed them to be the most informative. Further, we sought to minimize the number of outcomes in other to enhance the tables readability.

All the included articles were evaluated for risk of bias based on the Newcastle-Ottawa Quality Assessment Scale (NOS) developed for cross-sectional studies (Wells et al., 2014). When using the NOS, one gives points (in the form of stars) to each study based on the following three main categories: the sample selection, comparability between the groups, and outcome measures. Each article can receive from 0 to 10 stars, where a higher number indicates a lesser risk of bias. The process of data extraction and quality assessment were also performed in parallel and independently by two scholars (names omitted for anonymous review).

## 3 Results

### 3.1 Study selection

**Fig. 1:**
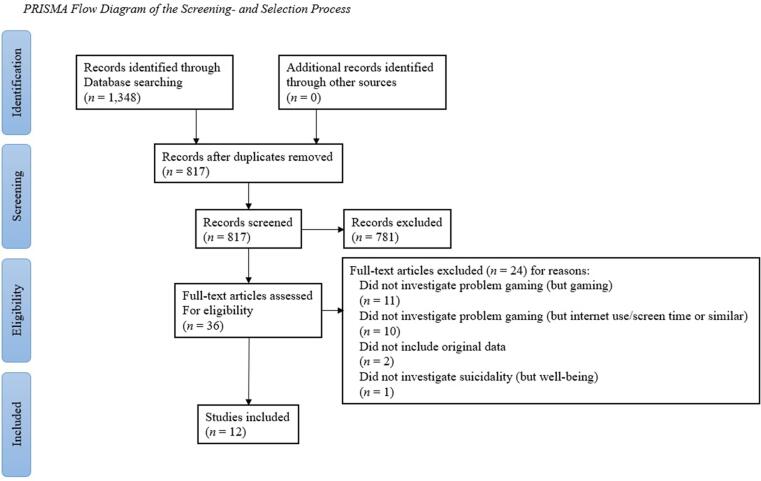
PRISMA Flow Diagram of the Screening and Selection Process.

Fig. 1 shows a flow chart of the selection process. The searches in the four databases resulted in a total of 1348 hits: 259 in PsycInfo, 448 in Web of Science, 288 in PubMed and 353 in EMBASE. No further articles were identified through the additional searches in Google Scholar (576 hits) or in the reference lists of included articles. After removing duplicates, 817 of the 1348 results remained. The first step in the selection process was to read through summaries and select relevant articles based on the inclusion and exclusion criteria. This step resulted in 36 articles that were read in full text. After reading the full texts, another 24 articles were excluded, hence 12 articles were finally included in the literature review (Bolat et al., 2021, Khalil et al., 2020, Kim et al., 2017, Lee and Ham, 2018, Merelle et al., 2017, Rehbein et al., 2010, Severo et al., 2020, Soares et al., 2020, Strittmatter et al., 2015, Wenzel et al., 2009, Yu et al., 2020). At full-text reading, the agreement between the two coders was 77%. In the cases of disagreement an agreement was reached by discussion. The disagreements were usually concerned with whether articles investigating gaming, but not problem gaming, should be included, in which only articles investigating problem gaming ended up being included. The following studies may appear to meet the inclusion criteria based on their titles: Ferguson and Smith, 2021, Förtsch et al., 2021, Gauthier et al., 2014, Messias et al., 2011, Mitchell et al., 2015, Rostad et al., 2021, and Teismann et al. (2014). Five of these seven studies were excluded because they did not measure problem gaming specifically, only gaming in general. The other two (Messias et al., 2011, Rostad et al., 2021), were excluded because they did not assess the association between gaming (or problem gaming) and suicidality but the association between total internet use/screen time (including gaming) and suicidality. The data extraction process resulted in a percentage agreement of 98%.

### 3.2 Descriptive characteristics of the included studies

Table 2 shows the characteristics of the included studies, and main findings. The included studies were published in the period 2009–2021. The sample sizes in the included studies varied from *n* = 92 (Bolat et al., 2021) to *n* = 44,610 (Rehbein et al., 2010) participants. One study was conducted in Africa (Khalil et al., 2020), two in South America (Severo et al., 2020, Soares et al., 2020), four in Asia (Jeong et al., 2018, Kim et al., 2017, Lee and Ham, 2018, Yu et al., 2020) and five in Europe (Bolat et al., 2021, Merelle et al., 2017, Rehbein et al., 2010, Strittmatter et al., 2015, Wenzel et al., 2009) (see Table 3).

**Table 2:** Study characteristics and main findings.

**Authors (year)**	**Country**	***N***	**Men (%)**	**Age M (SD)/range**	**PG (%)**	**Suicide outcome**	**Results**
Bolat et al. (2021)	Turkey	92	79.3	11.08 (2.1)	*NR*	Ideation	Positive association*
Jeong et al. (2018)	South-Korea	273	54.9	Adolescents	16.5	Ideation, plans, and/or attempts	*OR* = 1.31 (0.51–3.37) ^ns, 1^
Khalil et al. (2020)	Egypt	584	41.4	16.1 (1.2)	61.3	Ideation	Positive association^**^
Kim et al. (2017)	South-Korea	1,401	67.6 (PG), 70.1 (non-PG)	27.5 (8.3, PG), 33.6 (11.8, non-PG)	7.7	Ideation, plans, and attempts	*OR* suicide ideation = 3.03 (*CI* = 2.00–4.601)^***, 1^ *OR* suicide plans = 10.27 (*CI* = 5.56–18.96)^***, 1^ *OR* suicide attempts = 5.45 (*CI* = 2.94–10.10)^***, 1^
Lee & Ham (2018)	South-Korea	860	46.5	12–16	25.0	Ideation	*β* = 0.104^***, a^ *β =* 0.079^**, b^ *β =* 0.081^**, c^
Merelle et al. (2017)	The Netherlands	21,053	49.4	14.4 (1.3)	5.7	Ideation	*OR* = 2.12 (*CI* = 1.86–2.41)^**^ OR = 2.06 (*CI* = 1.75–2.42)^**, d^ *OR* = 2.28 (*CI* = 1.96–2.65)^**, e^
Rehbein et al. (2010)	Germany	44,610	51.3	15.3 (0.7)	4.5	Ideation	*OR* = 5.64 (*CI* = 3.53–8.99)^**, 1^
Severo et al. (2020)	Brasil	555	57.5	20.3 (5.4)	38.2	Ideation	*OR* = 2.49 (*CI* = 1.28–4.83)^**, 1^ *OR* = 0.73 (*CI* = 0.27–2.02)^ns, f^
Soares et al. (2020)	Brasil	6,026	44.7	16.5 (1.2)	*NR*	Ideation	*OR* (boys) = 1.08 (*CI* = 0.96–1.21)^ns^ *OR* (girls) = 1.26 (*CI* = 1.13–1.40)^***^ OR (girls) = 1.26 (*CI* = 0.93–1.30)^ns, g^
Strittmatter et al. (2015)	Estonia, Germany, Italy, Romania, and Spain	8,807	44.5	15.0 (1.3)	3.6	Ideation + attempts	*OR* = 4.23^***^
Wenzel et al. (2009)	Norway	3,405	51.1	16–74	2.2	Ideation	*OR* = 8.30 (*CI* = 2.50–22.65)^***, 1^
Yu et al. (2020)	China	1,066	56.5	13.0 (*NR*)	13.6	Ideation	*OR =* 2.78 (*CI* = 1.94–3.98)^***^ OR = 3.09 (*CI* = 2.10–4.54)^***, h^
*Notes. ^ns^*Not significant, *p < 0.05, ^**^p < 0.01, ^***^p < 0.001. ^1^*OR* were calculated for the purpose of the current review. *M* mean, *SD* standard deviation, *PG* problem gaming, *NR* not reported, *OR* odds ratio, *CI* confidence interval. ^a^Adjusted for: gender, grades, academic achievement, health, sleep disturbance, drug use, destructive behaviour/robbery, violent behaviour (inflictor), and depression, ^b^Adjusted for: ^a^ + source of stress, violence (victim), alienation, and teasing/harassment, ^c^Adjusted for: ^a^ + ^b^ + school location and satisfaction with school life, ^d^Adjusted for: age, gender, educational level, ethnicity, and family type, ^e^Adjusted for: the most significant covariates, ^f^Adjusted for: gender, age, education, family income, social anxiety, sleep, depression, and perceived academic achievement, ^g^Adjusted for: age and maternal educational level, ^h^Adjusted for: sex, age, mother‘s education level, father‘s educational level, perceived family financial condition, residence identity, family type, and living arrangements.

**Table 3:** Results from the Newcastle-Ottawa quality assessment.

	**Selection**				**Comparison**	**Outcome**		
**Authors (year)**	**Representativeness (Max:⋆)**	**Sample size (Max:⋆)**	**Non-respondents (Max:⋆)**	**Ascertainment of the exposure (Max:⋆⋆)**	**Comparable outcome groups/Controlled for confounding factors (Max:⋆⋆)**	**Assessment of outcome (Max:⋆⋆)**	**Statistical test (Max:⋆)**	**Total**
Bolat et al. (2021)	–	–	–	**	–	*	–	3*
Jeong et al. (2018)	*	–	*	**	–	*	–	5*
Khalil et al. (2020)	*	–	–	**	–	*	–	4*
Kim et al. (2017)	–	*	*	**	–	*	*	6*
Lee & Ham (2018)	*	–	–	**	**	*	–	6*
Merelle et al. (2017)	*	*	*	*	**	*	*	8*
Rehbein et al. (2010)	*	*	–	*	–	*	–	4*
Severo et al. (2020)	*	–	*	**	–	*	*	6*
Soares et al. (2020)	*	*	–	*	**	*	*	7*
Strittmatter et al. (2015)	*	*	–	**	–	*	*	6*
Wenzel et al. (2009)	*	*	*	*	**	*	–	7*
Yu et al. (2020)	*	*	–	*	**	*	*	7*

The age of the participants in the various studies varied from 7 to 74 years. Most studies used samples with adolescents (*n* = 10), one of these also included children in the sample (7–16 years) (Bolat et al., 2021), while two studies used adults (Kim et al., 2017, Wenzel et al., 2009). In terms of gender, the proportion of boys/men in the different samples varied from 41.4% (Khalil et al., 2020) to 79.3% (Bolat et al., 2021). The prevalence rates of problem gaming varied greatly in the various studies, ranging from 2.2% (Wenzel et al., 2009) to 61.3% (Khalil et al., 2020).

### 3.3 Problem gaming assessment

Two of the included studies used a diagnostic interview to map problem gaming (Jeong et al., 2018, Kim et al., 2017). The other 10 studies used self-report forms to obtain information about problem gaming. All the included studies used a categorical operationalisation of problem gaming, in which different measures with specific cut-offs for problem gaming were used, except for Bolat et al. (2021) who used a continuous measure of problem gaming. Two studies used time as a measure of problem gaming, in which more than 4 (Wenzel et al., 2009) and more than 5 (Soares et al., 2020) hours of gaming daily was defined as problem gaming. The remaining 10 studies used various measuring instruments/interviews based on an addiction framework to assess problem gaming. Strittmatter et al. (2015) did not assess problem gaming as such but instead assessed pathological internet use, in which they constructed a group consisting of individuals who were classified as pathological internet users and who at the same time reported to engage in gaming “frequently”.

### 3.4 Suicidality

None of the included studies included suicide as an outcome. Most studies used self-report forms to assess suicidal ideation and suicide attempts (*n* = 10), while two studies used diagnostic interviews (Khalil et al., 2020, Kim et al., 2017). All studies included measures of suicidal ideation, and three of the studies also included measures of suicide attempts (Jeong et al., 2018, Kim et al., 2017, Strittmatter et al., 2015). Kim et al. (2017) distinguished between suicidal ideation and suicide plans and reported on the association between problem gaming and these two variables, in addition to reporting the association between problem gaming and suicide attempts. Jeong et al., 2018, Strittmatter et al., 2015 combined suicidal ideation and suicide attempts and named these variables suicidality and suicidal behaviour, respectively, in which suicidality/suicidal behaviour was considered as present if any suicidal ideation or suicide attempts were reported the last year. Most studies (*n* = 11) operationalised suicidal ideation in a categorical manner, while Lee and Ham (2018) used a continuous measure. The three studies that included questions about suicide attempts used a categorical operationalisation as well (Jeong et al., 2018, Kim et al., 2017, Strittmatter et al., 2015).

### 3.5 Main findings

All the 10 studies that investigated the association between problem gaming and suicidal ideation (separately) found statistically significant, positive crude associations. In one study (Soares et al., 2020), the crude association was only significant for girls (not boys). One study investigated the association between problem gaming and suicide attempts (separately) and found a statistically significant, crude positive association for both outcomes (Kim et al., 2017). Jeong et al. (2018) who combined suicidal ideation and suicide attempts and named this variable suicidality, found a positive, but not statistically significant, association between problem gaming and “suicidality”. Strittmatter et al. (2015) who also combined suicidal ideation and suicide attempts in one variable called suicidal behaviour, found a statistically significant, positive association between problem gaming and “suicidal behaviour”.

Out of the 12 included studies, nine reported *OR* as an effect size (or enough information to calculate *OR*). In the current review both crude and adjusted *OR*s are reported. If several comparison groups were used (e.g., individuals with problem gaming being compared to individuals with “internet addiction” and individuals without problem gaming or “internet addiction”), the comparison to the most “normal” group is reported in the present review. In total 17 *OR*s reflecting the association between problem gaming and suicidal ideation, suicide plans, or suicide attempts are reported in the current review. These differed in magnitude, ranging from 0.73 (Severo et al., 2020, suicidal ideation in adjusted model) to 10.27 (Kim et al., 2017, suicide plans in crude model). *OR* is considered as an effect size, although its interpretation may be challenging as the magnitude of an *OR* depends on the rate of the dependent variable (Chen et al., 2010). Overall, it still has been suggested that *OR*s of 2.0, 3.0 and 4.0 indicate small, moderate, and large effect sizes, respectively (Ferguson, 2016). *OR*s below 2.0 might indicate a very small effect size, but such small ORs are hard to interpret (Ferguson, 2016). *OR*s below 1.0 means that the relationship is inverse, that the exposure variable in question is a protective factor. Following Ferguson (2016)‘s recommended cut-offs, five of the 17 *OR*s can be classified as very small, five as small, two as moderate, and five as large. Lee and Ham (2018) reported standardized betas as an indicator of effect size in one crude and two adjusted models. It has been suggested that standardized betas of 0.10, 0.30 and 0.50 indicate small, moderate, and large effect sizes (Cohen, 1988). The standardized betas reported in Lee and Ham (2018)‘s study were small or smaller than small (i.e., below 0.10). Two studies did not report effect sizes (or enough information to calculate effect sizes) (Bolat et al., 2021, Khalil et al., 2020).

Usually, the comparison groups consisted of individuals from the same sample as the problem gamers who did not experience problem gaming. In some cases (Severo et al., 2020, Wenzel et al., 2009, Yu et al., 2020), the comparison groups were limited to individuals who had played videogames (ever or the last year). In Strittmatter et al. (2015)‘s study, the comparison group consisted of individuals who were categorized as not experiencing “pathological internet use”.

All the 12 studies used a cross-sectional design. Five studies adjusted for possible confounders (Lee and Ham, 2018, Merelle et al., 2017, Severo et al., 2020, Soares et al., 2020, Yu et al., 2020), three of these found positive, significant associations between problem gaming and suicidal ideation upon such adjustment (Lee and Ham, 2018, Merelle et al., 2017, Yu et al., 2020). In Severo et al. (2020)‘s study the association between problem gaming and suicidal ideation was inverse when confounders were adjusted for, suggesting that problem gaming was a protective factor against suicidal ideation in this sample. This inverse association was not statistically significant. The confounders adjusted for included gender (Lee and Ham, 2018, Merelle et al., 2017, Severo et al., 2020, Yu et al., 2020), age (Merelle et al., 2017, Severo et al., 2020, Soares et al., 2020, Yu et al., 2020), ethnicity (Merelle et al., 2017), grades (Lee & Ham, 2018), academic achievement (Lee and Ham, 2018, Severo et al., 2020), educational level (Merelle et al., 2017, Severo et al., 2020), maternal educational level (Soares et al., 2020, Yu et al., 2020), paternal educational level (Yu et al., 2020), family type (Merelle et al., 2017, Yu et al., 2020), family financial situation (Severo et al., 2020, Yu et al., 2020), school location (Lee & Ham, 2018), residence identity (Yu et al., 2020), living arrangements (Yu et al., 2020), health (Lee & Ham, 2018), sleep disturbance (Lee and Ham, 2018, Severo et al., 2020), satisfaction with school life (Lee & Ham, 2018), depression (Lee and Ham, 2018, Severo et al., 2020), social anxiety (Severo et al., 2020), source of stress (Lee & Ham, 2018), violence (victim; Lee & Ham, 2018), alienation (Lee & Ham, 2018), teasing/harassment (Lee & Ham, 2018), drug use (Lee & Ham, 2018), destructive behaviour/robbery (Lee & Ham, 2018), and violent behaviour (inflictor; Lee & Ham, 2018).

### 3.6 Risk of bias assessment

The results from the risk of bias assessment are illustrated in Table 3. The assessment resulted in an 85% agreement between the two coders. The disagreements typically concerned what constituted satisfactory sample size and comparability between respondents and non-respondents. In the cases of disagreement, agreement was reached by discussion. The assessed risk of bias among included studies ranged from 3 to 8 stars. On average, the studies received a risk of bias score of 5.8 stars.

## 4 Discussion

In summary, the findings from the current review indicate that there is a positive association between problem gaming and suicidal ideation, although an inverse, non-significant association was observed in one study (Severo et al., 2020). The evidence concerning the association between problem gaming and suicide attempts was weaker as only three studies investigated this outcome (Jeong et al., 2018, Kim et al., 2017, Strittmatter et al., 2015). Two of these found a significant positive association (Kim et al., 2017, Strittmatter et al., 2015), while the third (Jeong et al., 2018) found a positive association that might have been significant if the study had a bigger sample size. No studies investigated the association between problem gaming and suicide. The effect sizes of the association between problem gaming and suicidal ideation/attempts varied between studies. Around half of the associations had very small or small effect sizes. The other half had moderate or large effect sizes. Relevant differences between studies that may explain differences in the observed effect sizes includes whether problem gaming was operationalised as a categorical or continuous variable and whether the measure of problem gaming was based on number of hours played or the presence of addiction symptoms. Only one study operationalised problem gaming as a continuous variable (Lee & Ham, 2018), the other studies used a categorical approach. The observed effect sizes in Lee and Ham (2018)‘s study appeared to be like the other effect sizes. Hence, whether problem gaming was treated as a continuous or categorical variable could not explain differences in effect sizes among the included studies. Two studies operationalised problem gaming based on hours played (Soares et al., 2020, Wenzel et al., 2009), the other studies used addiction-based measures of problem gaming. The effect size observed in Wenzel et al. (2009)‘s study was among the largest one among the included studies, which might suggest that hours played is a stronger indicator of suicidality compared to the presence of addiction symptoms. However, Wenzel et al. (2009)‘s study also differs from the other studies by being quite dated. Hence, the large effect size observed between hours played and suicidal ideation in Wenzel et al. (2009)‘s study might be a reflection of gaming and problem gaming being more uncommon at the time the study was conducted. The other study who used a measure of hours played, as opposed to addiction symptoms, found rather small effect sizes in which several of them were non-significant (Soares et al., 2020). Hence, it is possible that the association between problem gaming and suicidality is stronger when problem gaming is operationalised based on the presence of addiction symptoms rather than on hours played, but more research is needed to conclude on this issue. All included studies employed a cross-sectional design, and fewer than half of the studies adjusted for possible confounding variables. Because of this it is not possible to draw inferences concerning the causality/directionality regarding the observed relationship between problem gaming and suicidal ideation and attempts. The current study is, as far as we know, the first systematic review of the relationship between problem gaming and suicidality. Hence, there are no similar studies to compare the current findings with. However, the current findings are in accordance with the findings in a meta-analysis on the association between problematic internet use and suicidality which found that problematic internet use was associated with suicidal ideation (*OR* = 2.95; Cheng et al., 2018).

### 4.1 Limitations and strengths of the included studies

The most important limitation of the included studies is the lack of longitudinal designs. Further, the different studies differed in terms of the measures used to assess both problem gaming and suicidal ideation/attempts. The latter hampers the comparison of results across studies. Another important limitation with the included studies is that few assessed gaming characteristics (e.g., in terms of genre, single vs. multiplayer, and offline vs. online gaming) and none of the included studies adjusted for such gaming characteristics. This is an important limitation as gaming characteristics might act as moderators, mediators and/or third variables in the relationship between problem gaming and suicidality. Supporting the hypothesis that gaming characteristics might be important variables in the relationship between problem gaming and suicidality, the included studies that did provide some information regarding their sample‘s gaming characteristics found that those who experienced problem gaming differed from those who did not in terms of gaming characteristics (Jeong et al., 2018, Rehbein et al., 2010, Wenzel et al., 2009). Jeong et al. (2018) found that those who experienced problem gaming were more likely to play role playing-, shooter-, simulation-, arcade-, and online games and less likely to play sports games compared to non-problem gamers (not statistically significant differences, but the sample size was also quite small). Rehbein et al. (2010) found that World of Warcraft, online games, and PC-games were more popular among those who experienced problem gaming compared to those who did not. Also in Wenzel et al. (2009)‘s study were online games found to be more popular among individuals with problem gaming compared to those without. A strength of the included studies was that most of them had large sample sizes, in which 10 of the 12 studies had samples including more than 500 participants.

### 4.2 Limitations and strengths of the current review

The current review also has some limitations that should be noted. For one, a more thorough search for grey literature could have been conducted. Furthermore, some relevant studies may not have been identified in the current review due to the employed language restrictions and the exclusion of search words concerning self-injurious behaviour or self-harm (Klonsky et al., 2016).

The process used for conducting the research is one of the major strengths of the current review. Two coders independently selected studies, extracted data, and assessed risk of bias. The preregistration and the adherence to the PRISMA guidelines are other notably assets.

### 4.3 Implications

The results from the current review have several implications for future research. Future studies should aim to investigate the causality and mechanisms at play in the relationship between problem gaming and suicidal ideation/attempts. In particular, the relationship between game genre (e.g., sports games versus shooter games), problem gaming, and suicidality should be investigated by future research. It is reasonable to expect genre to affect the relationship between problem gaming and suicidality as both problem gaming and suicidality seem to be related to game genre (Gauthier et al., 2014, Rehbein et al., 2021). For instance, it could be speculated that more violent games might increase the likelihood of both problem gaming and suicidality. The current results also suggest a need for more studies on the relationship between problem gaming and suicide attempts and suicide. In addition, it could bring the field further forward if consensus in terms of assessment is reaches, as this would enable more direct and relevant comparisons across studies. Use of health registry data could also bring about more knowledge about the gaming problems – suicidality link.

Due to the many unanswered questions concerning the relationship between problem gaming and suicidality, few recommendations for clinicians and policymakers can be deduced based on the current results. Still, the current findings may suggest that clinicians working with individuals with problem gaming should consider inquiring about suicidal ideations and attempts.

## 5 Conclusions

In summary, the current review indicates that there is an association between problem gaming and suicidal ideation. The evidence concerning the association between problem gaming and suicide attempts is weaker as only three studies investigated this relationship. There is a need for more studies on the relationship between problem gaming and suicide attempts and suicide as well as studies using more stringent methodology (e.g., longitudinal designs). Clinicians working with individuals with problem gaming should consider inquiring about suicidal ideation and attempts.

## Funding

The data collection was funded by the Norwegian Competence Center for Gambling and Gaming Research. The funding source had no involvement in deciding the study design, data collection, analysis, interpretation of data, writing of the report, or in the decision to submit the article for publication.

### CRediT authorship contribution statement

**Eilin K. Erevik:** Conceptualization, Methodology, Formal analysis, Visualization, Writing – original draft, Writing – review & editing, Supervision, Project administration. **Helene Landrø:** Conceptualization, Methodology, Data curation, Formal analysis, Visualization, Writing – original draft, Writing – review & editing, Project administration. **Åse L. Mattson:** Conceptualization, Writing – review & editing, Supervision. **Joakim H. Kristensen:** Writing – review & editing. **Puneet Kaur:** Writing – review & editing. **Ståle Pallesen:** Conceptualization, Methodology, Writing – review & editing, Supervision, Funding acquisition.

## Declaration of Competing Interest

The authors declare that they have no known competing financial interests or personal relationships that could have appeared to influence the work reported in this paper.
